# Novel Thermosensitive Core–Shell Surface Molecularly Imprinted Polymers Based on SiO_2_ for the Selective Adsorption of Sulfamethazine

**DOI:** 10.3390/ma11112067

**Published:** 2018-10-23

**Authors:** Weihong Huang, Yujie Qing, Ningwei Wang, Yi Lu, Tianshu Liu, Tao Liu, Wenming Yang, Songjun Li

**Affiliations:** 1 School of the Environment and Safety Engineering, Jiangsu University, Zhenjiang 212013, China; whuang630@ujs.edu.cn (W.H.); 18851401862@163.com (Y.Q.); 2 Entry-Exit Inspection Quarantine Bureau, Zhenjiang 212008, China; niweiwang_2000@163.com (N.W.); luyi_2002@163.com (Y.L.); tianshuliu_2000@163.com (T.L.); taoliu_2017@163.com (T.L.); 3 Institute of Polymer Materials, School of Materials Science and Engineering, Jiangsu University, Zhenjiang 212013, China

**Keywords:** thermosensitive molecularly imprinted polymers, surface imprinting, sulfamethazine

## Abstract

In this research, a novel, sulfamethazine, thermosensitive, molecularly-imprinted polymer (MIP) with an obvious core–shell structure for the enrichment of sulfamethazine (SMZ), which involved temperature sensitive monomer N-Isopropylacrylamide, functional monomer methacrylic acid and cross-linking agents ethyleneglycol dimethacrylate (EGDMA) and N,N′-methylenebisacrylamide, was successfully compounded using the surface polymerization method. To ensure the best experimental group, we designed and compared three groups of controlled experiments of MIPs with different crosslinking agents. When the adsorption temperature was almost the lower critical solution temperature (LCST) of Poly(N-Isopropylacrylamide), the preparative MIPs showed outstanding adsorption capacity and specific identification to sulfamethazine. Moreover, this allowed the MIPs to better facilitate by combining the template molecules, as well as optimizing the imprinting factor. In addition, after 80 min, the adsorption of the MIPs leveled off and remained constant, and the adsorption quantity reached (a maximum of) at 8.1 mg·g^−1^.

## 1. Introduction

Sulfonamides (SAs), a type of synthetic antibacterial agent, were diffusely used in the prevention and treatment of various kind of infectious diseases by virtue of their antibacterial efficiency, low cost and other property advantages [1]. However, in recent years, SAs have been illegally used or even abused, causing antibiotics to potentially remain as hazards in the environment and food consumption [2]. Such food, to be absorbed by the human body for certain durations, will eventually threaten health, due to the accumulation/residual of SAs [3]. Meanwhile, people are obliged to pay more attention to their side effects, such as an increase of bacterial resistance, reduction of efficacy, allergic reactions, damage to the urinary system, as well as contamination of the environment.

In addition, the selected sulfamethazine (SMZ), which is one of SAs, due to its insolubility in water, is difficult to remove from a water environment using conventional methods [4]. At present, the inspection methods for SAs mainly comprise microbial detection [5], immunoassay [6,7], high performance liquid chromatography (HPLC) [8], thin-layer chromatography (TLC), gas chromatography (GC) [9], capillary electrophoresis (CE) [10], electrochemistry [11], liquid chromatography-tandem mass spectrometry (LC/MS) and so on. The above techniques are efficient and accurate, but some of them require cumbersome procedures to process and to enrich SMZ. Therefore, it is urgent to investigate an efficient, simple, economical and applied technique for the enrichment, separation and detection of SAs.

Molecular imprinting is a technique that can prepare polymers with selective adsorption to template molecules [12]. The prepared molecularly-imprinted polymers (MIPs) contain a binding site that exactly matches the size and shape of the template molecules. MIPs have been widely applied due to their selective identification and stability, such as solid phase extraction [13,14], sensors [15], drug delivery [16] and artificial antibodies [17,18,19]. In recent years, core–shell surface molecular imprinting technology is getting increasing attention. In the core–shell MIPs, the recognition sites are distributed on the surface of the nanoparticle shell, which not only resists the space mass transfer and allows the template molecules to be instantly adsorbed, but also facilitates the desorption of target molecules and improves the reusability of the imprinted material [20]. Li et al. reported a core–shell, thermosensitive, molecularly-imprinted nanomaterial for protein recognition [21]. Miao et al. manufactured the core–shell MIPs for the selective extraction of sulfonylurea herbicides residues [2]. In addition, the core–shell MIPs exhibit a satisfactory adsorption capacity in the water phase.

Nowadays, thermosensitive polymers (which are sensitive to temperature) have attracted more attention. Poly(N-isopropylacrylamide) (PNIPAM) was selected as a temperature-sensitive polymer. The notable feature of PNIPAM is that its lower critical solution temperature LCST is 32 °C [21]. When the temperature reached lower than LCST, PNIPAM showed a hydrophilic and loose structure. Conversely, when the solution temperature reached higher than LCST, PNIPAM showed a hydrophobic and crouched structure. This transformation was also reversible. Hence, the ability of MIPs to capture and release template molecules could be regulated by external temperature.

In this study, SMZ thermosensitive core–shell imprinted polymer microspheres based on SiO_2_ were prepared using the surface imprinting method. NIPAM, methacrylic acid (MAA), ethyleneglycol dimethacrylate (EGDMA) and N,N′-methylenebisacrylamide (MBA) were chosen to compose the imprinting shell. NIPAM was selected as the temperature-sensitive ingredient, which could swell and shrink as the temperature changes. MAA was the additional monomer due to the formation of the hydrogen bonding interactions with SMZ. Distinctively, both EGDMA and MBA were chosen as the crosslinking agents in order to obtain excellent adsorption for SMZ. The synthesized MIPs were characterized by fourier transform infrared (FTIR), static water contact angles and transmission electron microscope (TEM). Finally, the binding properties of MIPs for SMZ were evaluated by adsorption experiments.

## 2. Materials and Methods

### 2.1. Chemicals and Instruments

Sulfamethazine (SMZ; 99%), sulfamethoxazole (SMO; 99%), norfloxacin (NOR; 99%), methacrylic acid (MAA; 99%) and N-Isopropyl acrylamide (NIPAM; 98%) were purchased from Aladdin Reagent Co., Ltd. (Shanghai, China). MBA, ethylene glycol, EGDMA, γ-methacryloxypropyltrimethoxysilane (KH-570) and tetraethyl orthosilicate (TEOS; 98%) were supplied by Sigma Aldrich Chemical Co. (St. Louis, MO, USA). Glacial acetic acid (HAc), azobisisobutyronitrile (AIBN), acetonitrile, methanol, ammonia and ethanol were acquired from Sinopharm Chemical Reagent Co., Ltd. (Shanghai, China). Methanol (for HPLC) was obtained from Tedia Co., Inc. (Fairfield, OH, USA). All of the reagents were at least at analytical-reagent grade and were used without further purification.

Fourier transform infrared (FTIR) spectra were recorded on a Nicolet Nexus-470 FTIR spectrometer (USA) in the range of 450–4000 cm^−1^. The samples were mixed with KBr and pressed into a compact pellet. The morphology and structure of the microspheres were observed by using scanning electron microscopes (SEM, JEOL, JSM-7001-F, Tokyo, Japan) and transmission electron microscopes (TEM, JEOL, JEM-2100, Tokyo, Japan). The polymer content of MIPs and non-imprinted polymers (NIPs) was determined through a thermogravimetric analysis (TGA, NETZSCH, STA-449-C, Selb, Germany), in a temperature ranging from room temperature to 1000 °C under a nitrogen atmosphere with a heating rate of 10 °C/min.

### 2.2. Preparation of SiO_2_ Nanoparticles

Generally, in a typical experiment [22], deionized water (50 mL), ethanol (60 mL) and ammonia (10 mL, 3 wt%) were sequentially added to a 250 mL flask and vigorously magnetic stirred until the solution became homogeneous. Then, TEOS (5 mL) was slowly dripped into the above mixture, and the solution was reacted continuously at room temperature for 6 h. Afterwards, SiO_2_ microspheres were isolated by a centrifugation (8000 rpm, 5 min) and washed with deionized water and ethanol at least three times. Finally, it was dried in vacuum at 60 °C to a constant weight.

### 2.3. Synthesis of SiO_2_@MPS

The SiO_2_ (0.5 g, 8.3 mmol) was dispersed in toluene (100 mL) and ultrasonicated 15 min to form an average solution. Then, 4 mL KH-570 was dropped into the above solution and stirred vigorously. At last, the reaction took place under a nitrogen atmosphere at 65 °C for 24 h. The obtained SiO_2_@MPS were naturally cooled and collected by centrifugation (8000 rpm, 5 min), then rinsed with ethanol to dislodge unreacted substance and dried under vacuum at 60 °C for 6 h [23].

### 2.4. Preparation of Imprinted and Non-Imprinted Polymers

First of all, the template molecule SMZ (0.25 g, 1 mmol), functional monomers MAA (0.17 g, 2 mmol) and PNIPAM (0.34 g, 3 mmol) were dissolved in 50 mL of acetonitrile and ultrasonication, the solution was kept in a refrigerator at 4 °C for 10 h to obtain pre-polymerization. Then 300 mg of SiO_2_@MPS powder was dispersed in acetonitrile (50 mL) and ultrasonicated for 10 min, and EGDMA (15 mmol) was added to the above solution. Afterwards, both of the pre-assembled solutions were mixed and stirred under the protection of nitrogen for 10 min. After that, 0.12 g of AIBN was added into the mixture, the solution was reacted for 6 h at 50 °C, then the reaction was continued for 24 h at 60 °C The reaction product was washed with ethanol and dried to obtain SiO_2_@SMZ-MIPs. The SiO_2_@SMZ-NIPs were synthesized by the same method but without SMZ as the template molecule.

### 2.5. SMZ Adsorption Experiments

The adsorption kinetics experiments were used to study the adsorption saturation time and adsorption rate of MIPs and NIPs. The experiments were accomplished as follows: 10 mg MIPs and NIPs were respectively added to centrifuge tubes containing 5 mL of 0.3 mmol·L^−1^ SMZ solution. Then the tubes were placed in the thermostat and shaken at 35 °C. Afterwards, a small tube was taken at different time points and separated by centrifuging. The content of the sulfadimidine residues in the supernatant was determined by HPLC. Subsequently, the adsorption amount of the polymer to SMZ in each time period was calculated by using the following equation [24]:

$$q_{t}=\frac{{(C}_{0}{-C}_{t})\;V}{m}$$

where $q_{t}\;$(mg·g^−1^) is the adsorption amount of polymer to SMZ at time t; $C_{0}$ and $C_{t}$ (mg·L^−1^) represent the original concentration of SMZ and its concentration in the supernatant at time t; V (mL) was the volume of solution; and m (mg) was the mass of MIPs and NIPs.

Adsorption isotherm experiments were carried out: 10 mg of MIPs or NIPs were added to 5 mL of SMZ solution. The mixture was shaken for 5 h at 35 °C. After centrifugation, HPLC was utilized to determine the final concentration of SMZ in the supernatant. The SMZ’s adsorption capacity of MIPs and NIPs was calculated according to the following equation [25]:

$$q_{e}=\frac{{(C}_{0}{-C}_{e})\;V}{m}$$

where $q_{e}\;$(mg·g^−1^) is the saturated adsorption capacity of MIPs and NIPs; $C_{e}\;$(mg·L^−1^) represents the final concentration of SMZ in the supernatant when adsorbed equilibrium.

The imprinting factor (IF) was taken to evaluate the specificity of the prepared imprinted polymers; it was calculated according to the following equation:

$$\mathrm{IF}=\frac{Q_{\mathrm{MIP}}}{Q_{\mathrm{NIP}}}$$

where $Q_{\mathrm{MIP}}$ and $Q_{\mathrm{NIP}}$ represent the adsorption capacities of MIPs and NIPs to the template or the non-template, respectively.

A selective absorption experiment was applied to investigate the selectivity of MIPs and NIPs to target molecules or structural analogues, the typical procedures were as follows: 10 mg of MIPs/NIPs were dissolved in a solution containing 0.3 mmol·L^−1^ of SMZ, SMO or NOR. Then the solution was shaken at 35 °C for 5 h. The concentration of SMZ in the supernatant was determined after centrifugation.

## 3. Results and Discussion

### 3.1. Preparation of Molecularly-Imprinted Nanoparticles

**Figure 1:**
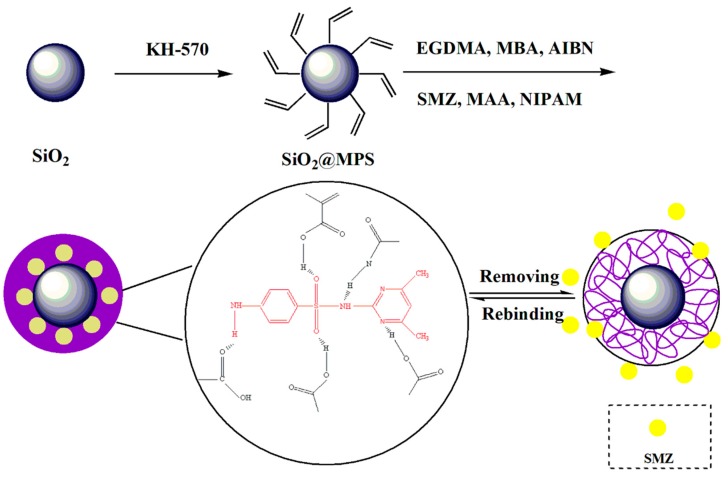
Schematic diagram of thermosensitive molecularly-imprinted polymers (MIPs) based on the surface of silica.

The process of synthesizing thermosensitive molecularly-imprinted polymers is shown in Figure 1. First, SiO_2_ was synthesized by the sol-gel method. Then, the vinyl was grafted on the surface of the SiO_2_ by reacting with MPS. Finally, the SiO_2_@MPS reacted with MAA, which made the nanoparticles easier to cover with polymer layers. In this paper, two cross-linking agents were selected, in which the amount of EGDMA could lead the polymer to form a certain rigid structure, so as to improve the reusability of MIPs. Meanwhile, MBA could optimize the hydrophilicity of the polymer [26,27] so that MIPs can be applied to the adsorption of SAs in a water environment, which expands the practical applications.

**Figure 2:**
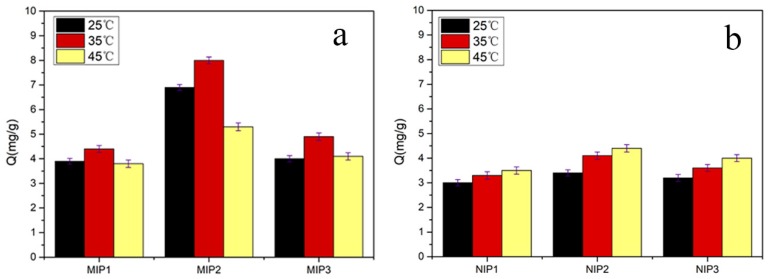
Adsorption capacities of three groups of MIPs (**a**) and non-imprinted polymers (NIPs) (**b**) at different temperatures (10 mg of MIPs/NIPs in 5 mL of 0.3 mmol·L^−1^ sulfamethazine (SMZ) solution for 5 h). Each Q value takes the average of three measurements, and the error bars represent the SD.

This section contributes to the study of the adsorption capacities of MIPs under influence factors of different types and proportions of crosslinkers. As shown in Table 1, three groups of MIPs/NIPs were prepared and the adsorption experiments carried out. Figure 2 displays the corresponding adsorption capacities of all kinds of MIPs and NIPs, among which MIP2 exhibited the greatest binding capacity for SMZ. The main reason for this may be excessive MBA that led to a particularly loose structure of MIPs, and the imprinting cavities were easily damaged or even failed when the template molecules were eluted. In addition, at three different temperature conditions, the absorption capacities of the MIPs to template molecules changed, which may be related to the phase transition behavior of temperature-sensitive functional monomers. At approximately 35 °C, the adsorption capacities of the imprinted polymers were relatively the largest, and the adsorption capacities decreased at 45 °C. This was because as the temperature rose, the hydrophobic interaction between the PNIPAM molecules and molecules in the thermoresponsive monomer was enhanced, and the hydrophilic effect weakened.

**Figure 3:**
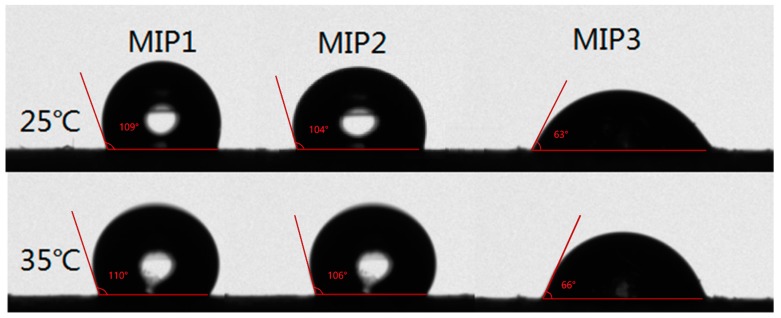
The test of static water contact angles of the different MIPs at 25 °C and 35 °C, respectively.

The results of the static water contact angles could exhibit the hydrophilicity or hydrophobicity of the imprinted polymers, which would be used as one of the criteria for screening the molecularly imprinted polymers with excellent hydrophilicity required by this experiment [28]. Three groups of imprinted materials were tested for static water contact angles at 25 °C and 35 °C, respectively. In Figure 3, it can be clearly seen that the order of the contact angles of the three polymers was MIP1 > MIP2 > MIP3. The smaller the water contact angle of the imprinted polymer, the better its hydrophilicity. Thus, it is concluded that the hydrophilicity of MIP3 was much better than MIP2, due to the higher content of MBA in MIP3 than in MIP2, effectively improving the surface hydrophilicity of the imprinted material. Although MIP3 was more hydrophilic than MIP2, the equilibrium adsorption capacities of MIP3 were smaller than MIP2, and MIP2 also has a certain degree of hydrophilicity. All further investigations of the extraction of SMZ were carried out, based on the above criteria, by using MIP2/NIP2 and simplifying the operation.

### 3.2. Characterization of Imprinted Nano-Microspheres

**Figure 4:**
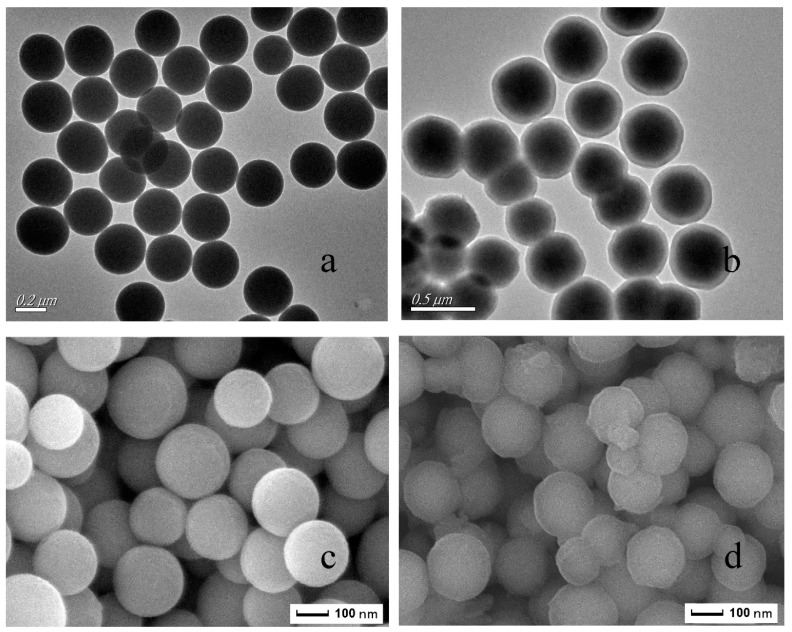
The transmission electron microscope (TEM) images of SiO_2_ (**a**), MIPs (**b**) and the scanning electron microscope (SEM) images of SiO_2_ (**c**), MIPs (**d**).

Figure 4 presents the SEM and TEM images of the SiO_2_ and MIPs. As shown in Figure 4a, the silica exhibits a uniform size, good dispersibility, and a particle size of about 250 nm. Figure 4b informs the core–shell structure of the microspheres, which demonstrates that the imprinted layer was successfully wrapped on the modified silica and that the imprinting layer was uniform in thickness, with the imprinting layer being about 15 nm. In contrast to Figure 4c,d, the surface of the silica was smooth, while the surface of the sphered silica was roughened by the inclusion of the blotting layer. In addition, the morphology and structure of the NIPs were not significantly different from those of the MIPs.

**Figure 5:**
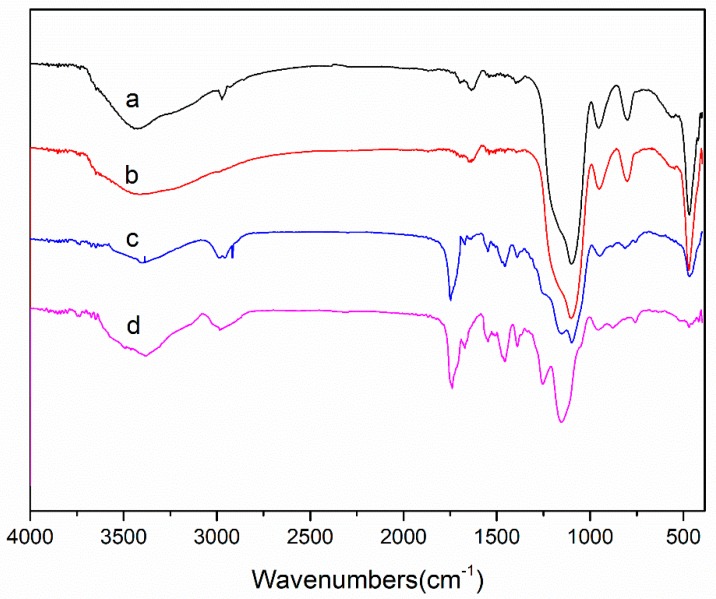
FT-IR spectra of the SiO_2_ (**a**), SiO_2_@MPS (**b**), MIPs (**c**), NIPs (**d**).

Figure 5 compares the FT-IR spectra of the SiO_2_, SiO_2_@MPS, MIPs, NIPs. In Figure 5a, the peak of 1099.2 cm^−1^ conformed with the Si–O–Si asymmetric stretching vibration, and the peaks above and under 468.4 cm^−1^ and 801.6 cm^−1^ could be attributed to the Si–O bending vibration and symmetrical stretching vibration, respectively. These strong peaks were typical SiO_2_ characteristic peaks. In comparison, Figure 5b shows the C=C absorption peak of the infrared spectrum of SiO_2_@MPS at 1646.6 cm^−1^, which indicated that the SiO_2_ was modified by KH-570. Figure 5c,d were approximately similar in shape, except for the peaks in Figure 5a,b, as well as the C=O vibrations of EGDMA at 1732.9 cm^−1^ and 1387.7 cm^−1^ peak and the C–H stretching peak. The amide I and amide II bands of NIPAM were observed at peaks 1637.7 cm^−1^ and 1545 cm^−1^, respectively, and 1456.2 cm^−1^ was marked as the –COO– stretching peak for MAA. The presence of these peaks indicated that the functional groups were present in each step of the synthesis. At the same time, a juxtaposition of Figure 5c,d informs the ability of the above characteristic peaks to prove that the imprinted layers were encapsulated on the surface of the spheres, and that the non-imprinted materials were also successfully prepared.

**Figure 6:**
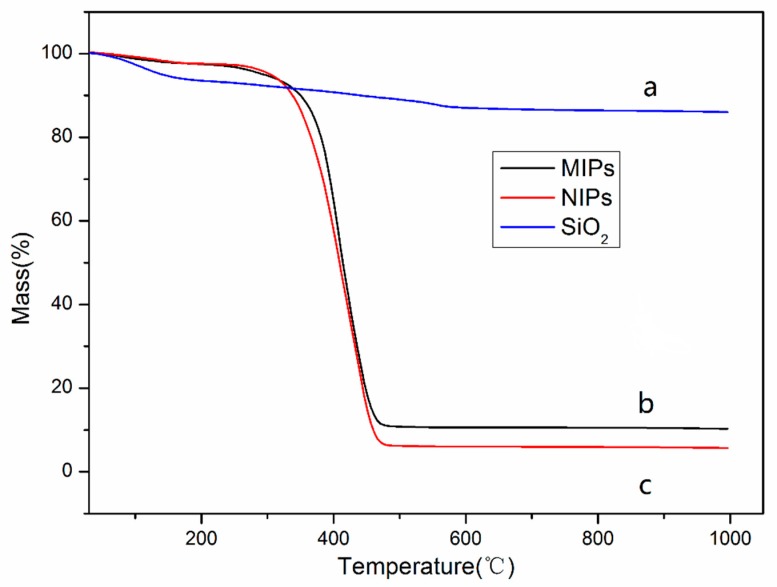
The thermogravimetric analysis (TGA) curves of SiO_2_ (**a**), MIPs (**b**), NIPs (**c**).

The thermogravimetric analysis (TGA) for SiO_2_, MIPs and NIPs are shown in Figure 6. It is clear to note that the weight of these substances exhibited varying degrees of loss as the temperature rose. Figure 6a shows a slight decrease, about 12%, mainly due to the evaporation of residual solvent on the surface of SiO_2_ at high temperature. The thermogravimetric profiles of MIPs and NIPs were roughly similar in shape, as the imprinted polymers that had eluted the template molecules were similar to those of the non-imprinted polymers. From 25 °C to 100 °C, the mass loss of MIPs and NIPs was not obvious, which was likely the result of the loss of residual moisture on the surface of the material. At 250 °C and 475 °C, the thermal gravimetric curves of MIPs and NIPs showed the most obvious drop with a mass loss of 87.2% and 90.6%, respectively. The mass loss at this temperature range could be attributed to the destruction of the polymer layer at high temperature. After about 450 °C, the thermogravimetric curves of the MIPs and NIPs began to flatten again. The mass loss in this temperature range can be attributed to the loss of carbonaceous materials and other substances in the silica.

### 3.3. Temprature Sensitivity Properties of MIPs

**Figure 7:**
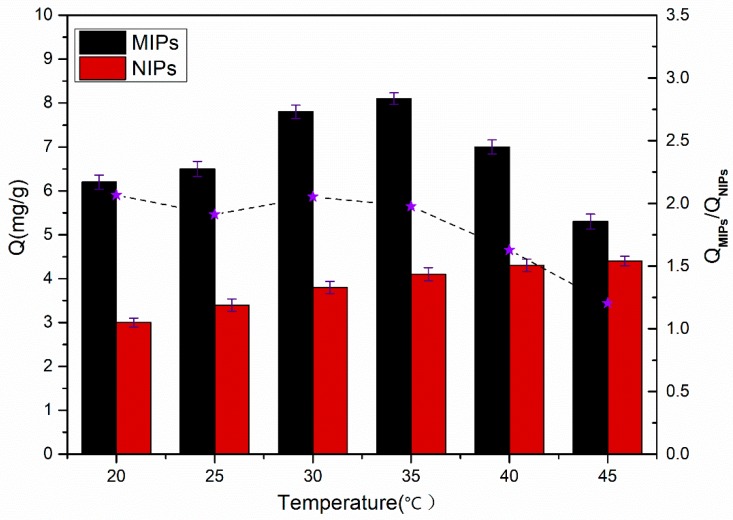
The adsorption capacities of MIPs and NIPs at different temperatures (10 mg of MIPs/NIPs in 5 mL of 0.3 mmol·L^−1^ SMZ solution for 5 h). Each Q value takes the average of three measurements, and the error bars represent the standard deviation (SD).

As shown in Figure 7, the adsorption amount of the template molecules (SMZ) on the MIPs and NIPs varies along with the temperature. Notably, the adsorption capacities of SMZ on MIPs first increased, before they decreased within the temperature range from 20 °C to 45 °C. The phenomenon depended on the volume change of molecular imprinted cavities, and the change was related to the existence of the temperature sensitive monomer NIPAM [29]. The Q value of the MIPs reached the maximum at 35 °C, because the shape and size of the imprinting pits were basically consistent with the template molecules. When the temperature was under 35 °C, the polymers were hydrophilic, and the imprinted cavities dilated. When the temperature was higher than 35 °C, the hydrogen bonding force was destroyed to a certain degree. As a result, the hydrophilicity of the polymer was weakened, and the imprinting cavities were too tight, which caused a decrease in the adsorption capacities with increasing temperature. However, the SMZ adsorption capacities of the NIPs varied as the temperature changed, with a different pattern from that of the MIPs. As the temperature increased, the adsorption capacities of the NIPs also slowly increased, because the increased hydrophobicity led to an increase in nonspecific adsorption. As shown, a variation in temperature can affect the spatial structure of the imprinted polymers, as well as the hydrophilicity and hydrophobicity, and can eventually impact the adsorption capacities of the MIPs of the target. Therefore, the next adsorption experiments were designed to be carried out at 35 °C.

### 3.4. Adsorption Experiments

#### 3.4.1. Adsorption Kinetics Experiments

**Figure 8:**
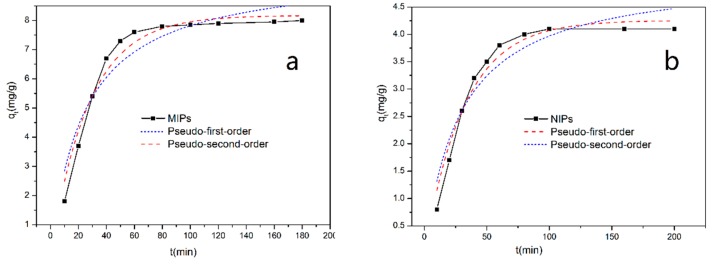
Adsorption kinetics fitting curves of MIPs (**a**) and NIPs (**b**) at 35 °C (10 mg of MIPs/NIPs in 5 mL of 0.3 mmol·L^−1^ SMZ solution).

At the optimized temperature, we investigated the adsorption properties of the MIPs and NIPs. Figure 8 reveals the adsorption kinetics fitting curves of MIPs (a) and NIPs (b) to SMZ at 35 °C. For the first time, the MIPs rapidly adsorbed SMZ at a rate of 81% of the maximum adsorption capacity at equilibrium. After 80 min, the adsorption capacities of the MIPs gradually stabilized and remained constant. This result indicated that the MIPs have a rapid adsorption rate due the many adsorption sites on the surface of nanoparticles. At the same time, the NIPs revealed a lower affinity, though much resembling the trend of the MIPs. This is because there were no specific sites existing in the NIPs, and the binding force to template molecules was also nonspecific.

In order to discuss the adsorption mechanism of SMZ on imprinted polymer, we fit the experimental data of the adsorption kinetics using a quasi-first-order kinetic model and a quasi-second-order kinetic model, respectively. The expressions for these two models are:

$$q_{t}{=q}_{e}{(1-e}^{{-k}_{1}t})$$

$$q_{t}=\frac{q_{e}^{2}k_{2}t}{{(1+q}_{e}k_{2}t)}$$

where $q_{e}$ (mg·g^−1^) and $q_{t}$ (mg·g^−1^) represent the adsorption capacities of MIPs on SMZ at adsorption equilibrium and at time t, respectively; $k_{1}$ and $k_{2}$ represent the adsorption rate constants of the quasi-first-order kinetic and the quasi-second-order kinetic, respectively.

Table 2 shows that the adsorption kinetics of MIPs at 35 °C can be well-fitted to the quasi-second-order kinetic model, and the standard deviation of the related parameters is shown in Table S1. The fitting correlation coefficient ($R^{2}$) was set at the largest, and the experimentally calculated adsorption $q_{e,\exp}$ was close to the theoretical $q_{e,\mathrm{cal}}$ value of the fitting result. This indicates that the adsorption of MIPs on SMZ belongs to chemisorption. However, the curves of NIPs were more in line with the quasi-first-order kinetic model ($R^{2}$ = 0.9392), which indicates that the force of the non-imprinted polymer on SMZ was physical.

#### 3.4.2. Adsorption Isotherms

**Figure 9:**
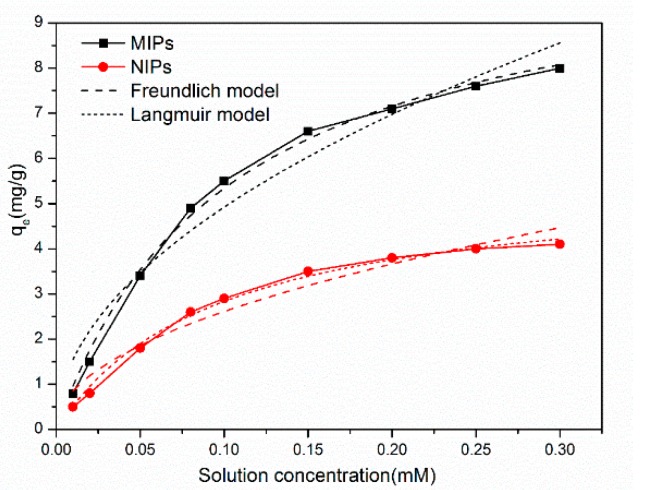
Adsorption isothermal fitting curves of MIPs and NIPs at 35 °C (10 mg of MIPs /NIPs in 5 mL SMZ solution with a concentration range of 0.02 to 0.3 mmol·L^−1^ for 5 h).

Figure 9 suggests that the adsorption capacities of MIPs/NIPs to SMZ were increasing as the templates initial concentration increased. We found that the Q value of MIPs on SMZ were all higher than those of NIPs. This can in turn be attributed to the imprinting effect.

In order to explore the adsorption process, we selected the Langmuir [30] and the Freundlich model to fit the experimental data. The expressions of the above two isotherm models were formulated as the following:

$$q_{e}=\frac{q_{\mathrm{mL}}k_{L}C_{e}}{{(1+k}_{L}C_{e})}$$

$$q_{e}{=k}_{F}C_{e}^{{1/n}_{F}}$$

where $q_{\mathrm{mL}}$ (mg·g^−1^) is the maximum adsorption capacity for monolayer adsorption; $k_{L}$ indicates the Langmuir constant; then $k_{F}$ and $n_{F}$ represent the Freundlich constants of adsorption capacity and adsorption strength, respectively.

The fitting parameters of the MIP and NIP adsorption data obtained from the above two isotherm models are shown in Table 3, and the standard deviation of the relevant parameters is shown in Table S1. The adsorption isotherms of MIPs at 35 °C coincided with the Langmuir model ($R^{2}$ = 0.9914), while the adsorption isotherms of NIPs were more in line with the Freundlich model ($R^{2}$ = 0.9888). This indicates that the adsorption mechanisms of MIPs were distinct from NIPs. The surface sites of the MIPs were uniform, and MIPs presented a monolayer adsorption to SMZ. Inversely, the adsorption sites were not evenly distributed on the surface of the NIPs. In conclusion, the selectivity of the MIPs was further confirmed.

#### 3.4.3. Rebinding Specificity

**Figure 10:**
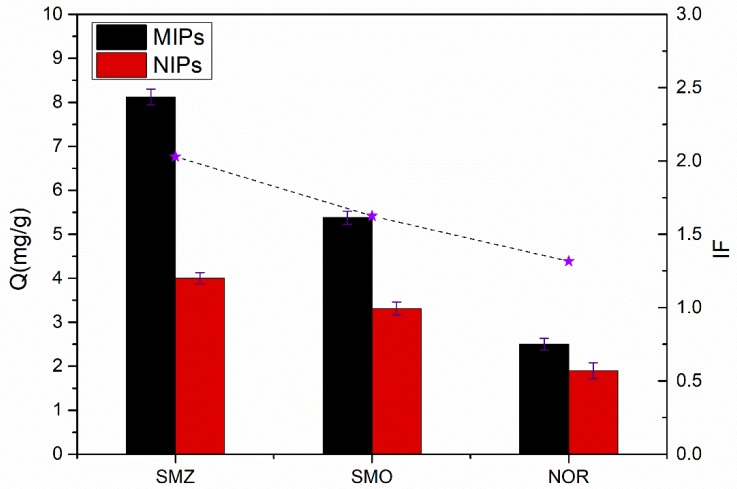
The binding amounts of MIPs and NIPs for SMZ, sulfamethoxazole (SMO) and norfloxacin (NOR) (10 mg of MIPs/NIPs in 5 mL solution containing 0.3 mmol·L^−1^ of SMZ, SMO or NOR for 5 h). Each Q value takes the average of three measurements, and the error bars represent the SD.

Figure 10 shows the adsorption capacities of the MIPs and NIPs for SMZ, its structural analogue SMO, and the structurally dissimilar materials NOR. As shown in Figure 10, the adsorption capacities of MIPs to SMO, as well as those of the NOR, were lower than SMZ, indicating that the MIPs had nearly no selective adsorption to SMO and NOR, whereas the target molecule SMZ had a comparatively higher affinity for the imprinted polymers. The result indicates that the conformational memory effect of the imprinting cavities was an important factor the imprinting efficiency. MIPs slightly adsorbed SMO, owing to the fact that the molecular structure of SMO also contained –S(=O)^2−^ groups. The adsorption capacity of MIPs to NOR was low due to the fact that the molecular structure and size of NOR were far from the imprinting cavities and thus did not easily form functional forces with functional monomers. However, the binding amounts of NIPs to these three substances were similar. This is likely a result of the fact that NIPs had no imprinting sites on the surface, and that their adsorption forces on SMZ, SMO and NOR were nonspecific and physical in nature.

#### 3.4.4. Reusability

**Figure 11:**
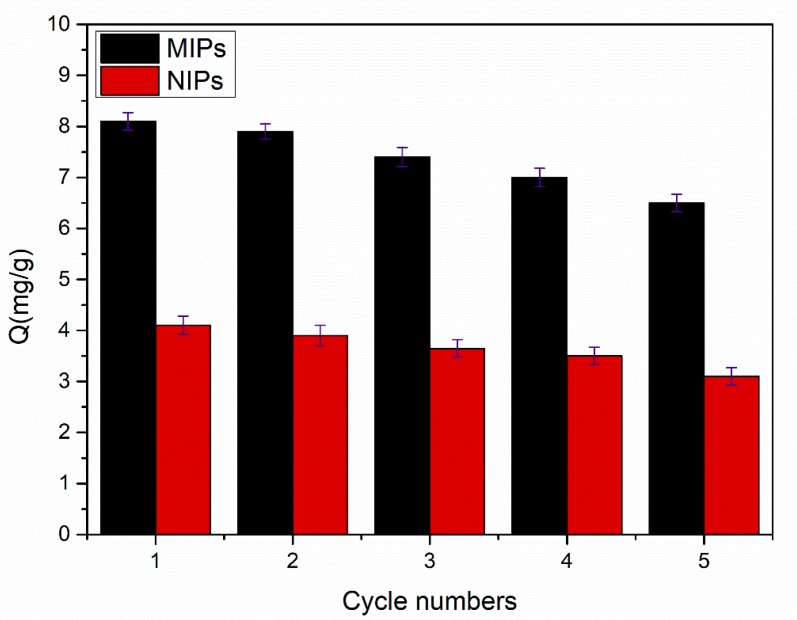
The recycling of MIPs and NIPs (10 mg of MIPs / NIPs in 5 mL of 0.3 mmol·L^−1^ SMZ solution for 5 h). Each Q value takes the average of three measurements, and the error bars represent the SD.

In order to realize the stability and the reusability of the imprinted polymers, the regeneration experiments were carried out at the temperature of 35 °C with a 0.3 mmol·L^−1^ SMZ aqueous solution. First, MIPs and NIPs were subjected to isothermal adsorption for 5 h, then methanolic:acetic acid (9:1, *v*/*v*) was added and the target substance SMZ was eluted by ultrasonic oscillation. The process was repeated five times. Figure 11 clearly indicates that the MIPs decreased the amount of SMZ adsorption by nearly 19.8% after five cycles of experiments. This might be a consequence of the fact that the spatial structure of the imprinted holes was destroyed to a certain degree by the action of the ultrasound, and that the target in the imprinted site might not be completely eluted [31].

## 4. Conclusions

Thermosensitive core–shell MIPs were prepared by copolymerization, based on the surface of SiO_2_@MPS nanoparticles in the SMZ solution. The influence of temperature on the adsorption capacities of the imprinting materials was also discussed, and the results provided an explanation of the fact that the reversible volume change of the MIPs between the expanded and the collapsed phases can occur as the temperature changes. The MIPs exhibited a favorable recognition effect for SMZ, and the maximum adsorption was 8.1 mg·g^−1^, which was much higher than that of the NIPs. Meanwhile, the imprinting factor of the MIPs for SMZ could reach 2.03, which was higher than that of SMO (IF = 1.62) and NOR (IF = 1.31), which indicated the high selectivity of MIPs for SMZ. Moreover, the reusability of MIPs/NIPs was investigated through five adsorption–desorption cycles, as a result, the prepared MIPs/NIPs showed excellent stability. Consequently, the adsorption and desorption of MIPs to SMZ was able to be regulated by changing the temperature. In short, the proposed core–shell imprinted polymers could be used to effectively extract SMZ from actual samples. It is expected that the MIPs could be a promising material for environmental detection.

## Figures and Tables

**Table 1:** Synthetic formula of MIPs.

Composition (mmol)	MIP1	MIP2	MIP3
SMZ	1	1	1
MAA	2	2	2
PNIPAM	3	3	3
EGDMA	15	10	5
MBA	0	5	10

**Table 2:** Adsorption kinetics fitting parameters of MIPs and NIPs towards SMZ.

Kinetic Models	Parameters	MIPs	NIPs
Pseudo-first-order	q_e,cal_ (mg·g^−1^)	9.715	4.255
	k_1_ (min^−1^)	0.0043	0.0314
	R^2^	0.8163	0.9392
Pseudo-second-order	q_e,cal_ (mg·g^−1^)	8.172	5.120
	k_2_ (min^−1^)	0.0363	0.0067
	R_2_	0.9297	0.8324
Experimental data	q_e,exp_ (mg·g^−1^)	8.091	4.124

**Table 3:** Adsorption isothermal fitting parameters of MIPs and NIPs towards SMZ.

Isotherm Models	Parameters	MIPs	NIPs
Langmuir	k_L_ (L·mg^−1^)	0.9661	1.0322
	q_mL_ (mg·g^−1^)	10.862	5.578
	R^2^	0.9914	0.8968
Freundlich	k_F_	15.6891	8.0438
	n_F_	1.9863	2.0468
	R^2^	0.9072	0.9888

## Supplementary Materials

The following are available online at http://www.mdpi.com/1996-1944/11/11/2067/s1, Table S1: the standard deviation of the parameters in each fitting models.
