# Supplementary material for: Novel Thermosensitive Core–Shell Surface Molecularly Imprinted Polymers Based on SiO2 for the Selective Adsorption of Sulfamethazine
Source: Materials (Basel). 2018 Oct 23;11(11):2067. doi: 10.3390/ma11112067 (PMC6266568; doi:10.3390/ma11112067)
Supplement: Supplementary file 1 [file materials-11-02067-s001.pdf]

materials

Supporting information

Novel Thermosensitive Core-Shell Surface Molecularly Imprinted Polymers Based on SiO_2 for the Selective Adsorption of Sulfamethazine

Weihong Huang [1], Yujie Qing [1], Ningwei Wang [2], Yi Lu [2], Tianshu Liu [2], Tao Liu [2], Wenming Yang [3,*] and Songjun Li [3,*]

[1] School of the Environment and Safety Engineering, Jiangsu University, Zhenjiang 212013, China; whuang630@ujs.edu.cn (W.H.); 18851401862@163.com(Y.Q.)
[2] Entry-Exit Inspection Quarantine Bureau, Zhenjiang 212008, China; niweiwang_2000@163.com (N.W.); luyi_2002@163.com (Y.L.); tianshuliu_2000@163.com (T.L.); taoliu_2017@163.com (T.L.)
[3] Institute of Polymer Materials, School of Materials Science and Engineering, Jiangsu University, Zhenjiang 212013, China
* Correspondence: ywm@ujs.edu.cn (W.Y.); lsjchem@ujs.edu.cn (S.L.); Tel.: +86-511-8879-1919 (W.Y.)

Received: 02 August 2018; Accepted: 12 September 2018; Published: date

Table S1 the standard deviation of the parameters in each fitting models.

Kinetic models.	Parameters	Standard deviation	
		MIP	NIP
Pseudo-first-order	q_e	0.5953	0.1135
	k_1	0.0011	0.0027
Pseudo-second-order	q_e	0.2006	0.3652
	k_2	0.0031	0.0019
Langmuir	k_L	0.0764	0.0905
	q_{mL}	0.3429	0.1901
Freundlich	k_F	0.5738	0.3295
	n_F	0.2145	0.2323
